# Raman imaging of molecular groups in the wavenumber silent region

**DOI:** 10.1007/s00216-025-06029-1

**Published:** 2025-08-13

**Authors:** Constanze Schultz, Jürgen Popp

**Affiliations:** 1 Leibniz Institute of Photonic Technology (Leibniz-IPHT), Member of Leibniz Health Technologies, Member of the Leibniz Center for Photonics in Infection Research (LPI), Albert-Einstein-Straße 9, 07745 Jena, Germany; 2 Institute of Physical Chemistry (IPC) and Abbe Center of Photonics (ACP), Member of the Leibniz Center for Photonics in Infection Research (LPI), Friedrich Schiller University Jena, Helmholtzweg 4, 07743 Jena, Germany

**Keywords:** Raman tags, Alkyne, Nitrile, Isotope labelling, Application, Molecular detection

## Abstract

Raman imaging in the wavenumber silent region emerged around 15 years ago as a powerful tool for visualizing biomolecules and synthetic compounds in complex environments with minimal spectral and biological interference. Since then, the field has advanced from simple proof-of-concept studies using available tags to the rational design of highly efficient Raman labels with sharp silent region signatures, now applied to real biological and biomedical questions. This review traces the evolution from the versatility of label-free Raman to the increasing relevance of labeled strategies, emphasizing how tag design influences functionality, application, and impact. We highlight recent progress in both the synthesis and deployment of tags tailored for specific cellular targets and processes and discuss the emerging need for labeled strategies to meet the demands of sensitivity, multiplexing, and biocompatibility in complex systems. Through this design-to-application perspective, the review provides a comprehensive overview of the current capabilities and significant applications, and identifies key future directions to fully exploit the potential of silent region Raman imaging.

**Graphical Abstract:**

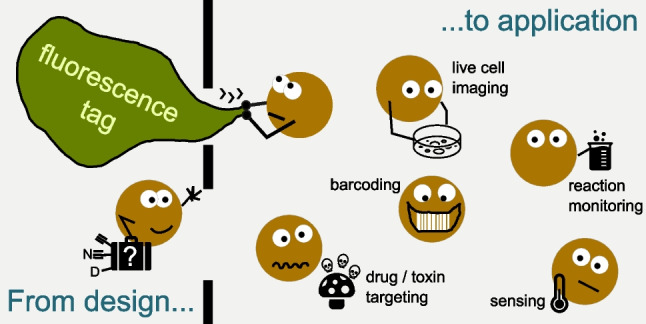

## Introduction

Imaging methods have become an essential component of scientific research, serving as key tools and strategies to address a wide range of research questions. In analytical and bioanalytical chemistry, often the question on the molecular presence persists, concluding either from sample structure to composition (morphochemistry) or, vice versa, elucidating the presence of a specific target at a certain location (targeting). The available palette of technologies for spatially addressing molecular composition is diverse, encompassing mass spectrometry, radionuclide imaging, electron microscopy, magnetic resonance imaging (MRI/NMR), fluorescence microscopy, vibrational imaging (e.g., Raman and infrared spectroscopy), among others. Each method offers unique advantages and specific limitations, which guide their application based on the molecular specificity, sensitivity, and required resolution. Especially when tracing minute amounts of substances in complex environments, specificity and sensitivity become critical parameters, as they determine the limit of detection of a characteristic as well as its separability from and uniqueness to others. Consequently, maximizing both specificity and sensitivity is central to ongoing research on new imaging concepts.

## Raman spectroscopy — a tool with many uses

**Fig. 1:**
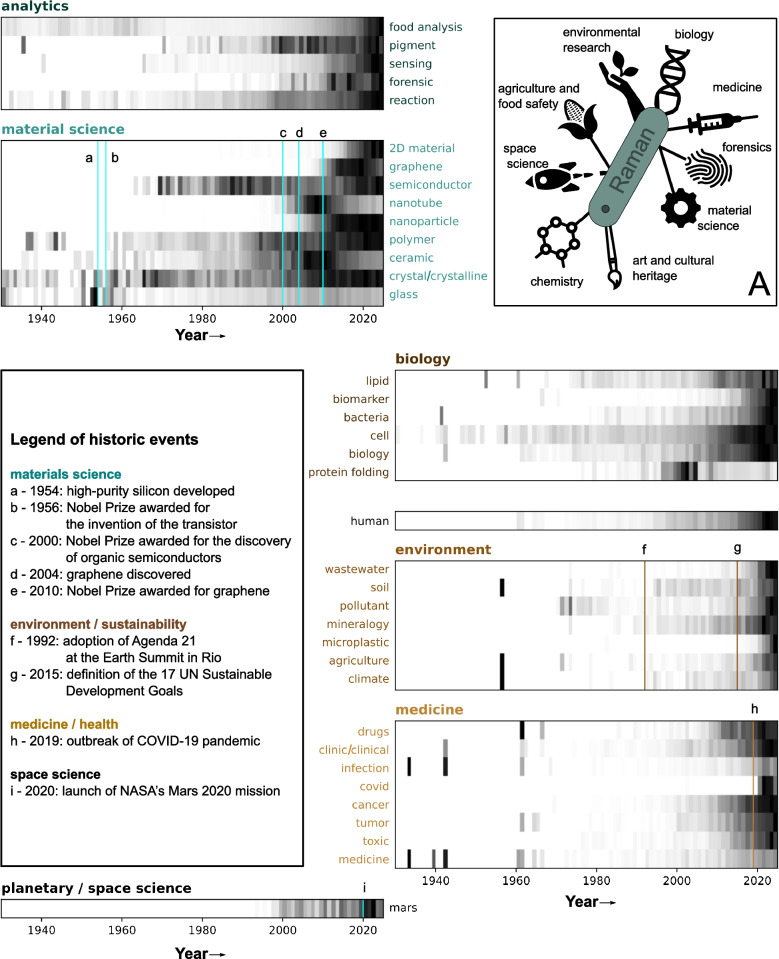
The fields of application of Raman spectroscopy are broad (inset A). The heatmaps visualize the number of hits within the OpenAlex database for Raman + keyword in the title and abstract search relative to the number of papers with only Raman being present. Normalization was performed on a per-keyword basis. Lines indicate important events as can be taken from the figure legend

Since the discovery of the Raman effect by Chandrasekhara Venkata Raman in 1928 [1–3], and its continued development as a spectroscopic characterization method, vibrational Raman spectroscopy (in the following called Raman) has evolved into a versatile analytical tool applied across numerous disciplines (Fig. 1A). The power of Raman spectroscopy primarily lies in its exceptional specificity. When light interacts with a sample, small fractions of light can scatter inelastically, carrying information about the sample’s molecular structure. This information arises in the case of vibrational Raman spectroscopy from the consumption or release of energy associated with molecular bond vibrations. Against this backdrop, the recognition that wavenumber shifts between incident and Raman-scattered radiation coincide with known vibrational infrared transitions [3], prompted intense investigations in the use of this new source of secondary radiation across various organic molecules and inorganic materials. These early investigations yielded profound insights into the shape and features of Raman spectra and the respective vibrational frequencies in dependence on structural aspects such as the valence state of substituent groups and bond order, atomic combinations, molecular geometry, including rotational and structural isomerism (cis–trans), as well as spatial structure [4–6]. Allocating all these scattered frequencies eventually provides a fingerprint of the focal spot’s molecular composition, allowing even for the distinction of different peculiarities of the same molecular class (e.g., different amino acids [7]).

The analytical value of Raman spectroscopy for structural elucidation has endured to this day. Within chemical sciences, Raman is still an inevitable tool for structural characterization of newly synthesized compounds but has also gained attention in the classical analytical sense for reaction monitoring [8, 9], as a sensing tool for substance detection or industrial quality control [10, 11]. However, the analytical specificity enables applications far beyond classical chemistry. To better visualize the diversity of Raman’s fields of use, we searched the titles and abstracts of papers within the OpenAlex database [12] by combinations of “Raman” and (an)other keyword(s) and visualized the number of found papers relative to the ones with only the keyword “Raman” by years (Fig. 1). We identified 37 keywords worth evaluating. Clustering them by overarching topics provided a comprehensive overview. This visualization clearly shows that while some topics have evolved gradually over time, others strongly correlate with historical events, highlighting the broad applicability, actuality and growing scope of Raman spectroscopy. Following its use in the analysis of simple molecules, more complex mixtures have become central, for example in art and cultural heritage research, where Raman spectroscopy helps determine the chemical composition of paints and pigments [13] and material compositions, providing insight into the age, geographical origin, and cultural context of artifacts [14, 15]. In a broader sense, materials research has always been of interest for Raman spectroscopy, progressing from the research in glass and crystals via ceramics to polymer research and further on to nanoparticles (Fig. 1). Hot topics in material research involved semiconductors starting close after the invention and Nobel Prize of the transistor with a further peak in recent years. Also, the research in nanostructured materials shows clear peaks. As such, we consider carbon nanotubes, graphene following its Nobel Prize in 2004, and other 2D materials emerging in recent years. Emerging applications cover the analysis of strain, doping, defects and purity of nanomaterials [16], insights into their layer structure [17], as well as investigations of the binding conditions in new smart polymer materials [18, 19], among others.

Spontaneous Raman scattering, along with its stimulated counterparts, coherent anti-Stokes Raman scattering (CARS) and stimulated Raman scattering (SRS), has greatly benefited from the advent of powerful laser sources in the mid-twentieth century. These advancements have led to the development of commercially available, rather than exclusively home-built, systems, facilitating non-destructive and label-free analysis of a wide range of samples, operating with ease in aqueous environments. These characteristics make Raman especially interesting for biological and medical research but do not limit it exclusively to it. In biological research, Raman spectroscopy is, e.g., used to analyze plants, microorganisms, and cells, with growing emphasis on live-cell imaging through faster nonlinear techniques. Research has focused on biomarker identification and recognition, as well as on the secondary structure elucidation of proteins, particularly in the 2000 s and relevant to biomedical studies, such as for Alzheimer’s disease [20, 21].

The transition from biological to medical research is often seamless. Tracking the progression of the keyword “human” reveals a growing emphasis on both medical and environmental applications in recent years. In the medical domain, Raman spectroscopy has emerged as a powerful tool for distinguishing between healthy and diseased tissues, the assessment of the grade of infection or disease state, as well as identification of pathogens and antibiotic resistance testing [22, 23]. The area of medical research is thereby inevitably linked to advances in the fields of AI, which aim to establish Raman microspectroscopy as a histopathological tool [24]. In parallel, the development of compact, portable Raman spectrometers has spurred activity in forensic science. Applications include, e.g., the differentiation of body fluids, analysis of blood properties such as age or donor identification, questioned document analysis, detection of toxic substances, and classification of gunshot residue particles [25–27].

Since the adoption of the UN Agenda 2030 and the articulation of global climate goals, environmental research has become a rapidly growing field. Raman spectroscopy now plays an essential role in assessing crop health and sustainable agriculture [28], ensuring food safety [29], identifying alternative food and energy sources [30], and detecting pollutants in air, water, soil, and ice. As such, high emergence is currently addressed to the critical issue of microplastics [31, 32].

## Small molecules, big challenges

While this overview highlights Raman spectroscopy’s broad potential, Raman scattering remains an inherently rather inefficient process. As only one in a million photons is actually scattered inelastically, the Raman effect and thus Raman’s sensitivity is very weak. Intensity-enhanced technologies, such as surface-enhanced Raman scattering (SERS) or tip-enhanced Raman scattering (TERS) using local plasmon resonances yield a much better sensitivity, enabling trace detection and/or improved spatial resolution, but require active metallic nanostructures that may not be compatible with all samples.

In a biological context, Raman spectroscopic techniques often compete directly with fluorescence imaging. Fluorescence excels particularly when intrinsic fluorophores, such as flavins, porphyrins, and carotenoids, are present, which enables a sensitive, highly specific, and label-free detection that often surpasses the need for Raman spectroscopy in such cases. Fluorescence detection also finds broad application in multiplexed imaging, where simultaneous detection of multiple fluorophores allows for colocalization analyses, visualization of distinct cellular substructures [33] or barcoding purposes [34], provided that emission and detection windows are well separated. However, the inherently broad absorption and emission bands of fluorophores typically limit specificity to 3–4 well-separated dyes to avoid spectral crosstalk and bleed-through. In some cases, fluorescence lifetime imaging (FLIM) can bypass these spectral constraints by distinguishing fluorophores based on their lifetimes [28], but its technical complexity often limits its accessibility.

Consequently, for custom multiplexing or to render non-autofluorescent molecules detectable by fluorescence imaging, a strategic selection of fluorophore systems is essential. Effective fluorophores typically rely on extended π-electron systems, whose native prevalence decreases significantly with decreasing molecular size. As many molecules of interest in biological sciences possess a molecular weight below 1000 g/mol and can thus be considered as small, the use of fluorescence spectroscopy is limited. Although commercially available or custom-designed fluorescent tags can compensate for the lack of intrinsic fluorescence, the exceptional sensitivity of fluorescence microscopy is often offset by the uncertainty as to whether the native function of the investigated molecules is retained. This concern arises because external fluorescent tags can be relatively bulky, potentially interfering with biological activity [35].

While Raman spectroscopy offers excellent specificity, its practical sensitivity is limited for the direct analysis of small molecules within complex biological environments, unless specialized enhancement techniques such as surface-enhanced Raman scattering (SERS) or optimized Raman tags are employed. In some cases, where accumulation spots are very distinct or band patterns appear that are highly enriched in comparison to the surrounding environment, a distinction via the fingerprint region of the vibrational spectrum is possible. Characteristic examples include the C = C stretching vibration (retinoic acid derivatives [36]) or breathing vibrations of aromatic frameworks [37]. However, most of the prevalent bonds in typical organic molecules (C-H, C–C, N–O, P-O, S–O, C-N, C-O) are not very distinct, which makes a differentiation within a typical biological environment challenging or at least expensive by fingerprint Raman spectroscopy.

Raman groups that operate in the wavenumber silent region of the vibrational spectrum can be a fruitful bridge in these cases, combining the broad scope and specificity of Raman imaging with the functional advantages of external labels, such as multiplexing and customizable probe design, which is commonly seen in fluorescence microscopy. This review will thereby focus exclusively on silent region Raman tags that could be employed for spontaneous and stimulated Raman scattering. Tag design strategies and applications correlated with SERS imaging will be excluded on purpose within this review.

## Raman tags — from design to applications

Although vibrational groups with bands in the otherwise wavenumber silent region of the vibrational spectrum were known earlier [5, 38], Raman tagging truly gained momentum around 2010, driven by pioneering studies from groups such as those of Sodeoka and Min. They developed very efficient triple-bond tag types as external labels as outlined later. Therefore, Raman tags have evolved as a vital strategy in small molecule imaging with a scope almost as diverse as that of untagged Raman scattering. To the best of our knowledge, applications span across many of the bio-related areas, including their use for environmental, biological, biomedical, and pharmaceutical aspects, while the tags’ use for pure material science purposes is currently limited. In this review, we systematically examine the key properties that define effective Raman tags within the wavenumber silent region, along with the types of applications where these tags are most impactful. While it is evident that no single property alone determines the suitability of a molecular group or structural element as a Raman tag, we classified the applications by the property which is most decisive for successful use in the given case.

### Raman tags are orthogonal

**Fig. 2:**
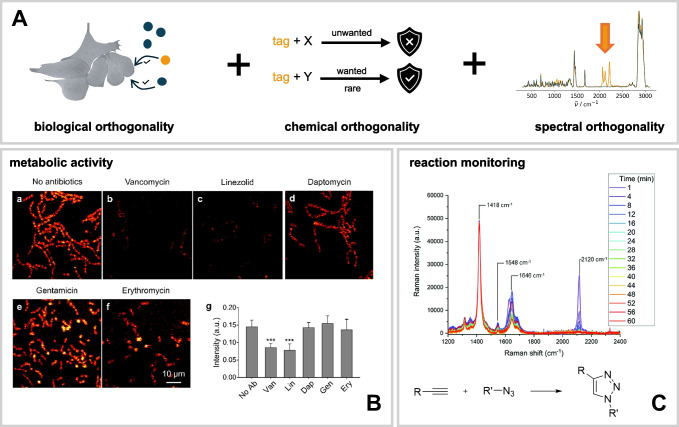
The spectral orthogonality of Raman tags (**A**) enables clear distinction of the tagged substance from its surroundings. This property has been applied in studies of newly synthesized compounds, respectively, in monitoring bacterial activity (**B**) and reaction monitoring (**C**). In panel **B**, *E. faecalis* 31970 is cultivated in glucose-d_7_ for 1 h with or without antibiotics [39]. If the bacterium is resistant to the antibiotic, it remains metabolically active and incorporates the isotopic label into its biomass. In contrast, if the bacterium is susceptible to the antibiotic, its metabolic activity is significantly reduced, resulting in little to no detectable deuterium signal. **B** is reprinted with permission from Ref. [39], Copyright 2018, American Chemical Society). **C** When a Raman tag is formed or cleaved during a reaction, this transformation can be detected spectroscopically as shown here for the alkyne-azide cycloaddition (spectrum reproduced from Ref. [40] with permission from the Royal Society of Chemistry). The cell icon within panel **A** by DBCLS https://togotv.dbcls.jp/en/pics.html is licensed under CC-BY 4.0

An important feature concerning the effectiveness of a label is its distinguishability from the surrounding environment (Fig. 2A). The term “environment” can be interpreted in two ways: firstly, the chemical or biological background matrix in which the target molecules are embedded, and secondly, the spectral background against which Raman signals must be distinctly identifiable. In biological sciences, this native background consists of other biomolecules with their most prominent bond types stated above. Thus, a tag would obey a trackable bond that is not common or not common in high numbers to the surrounding matrix while preserving native function (biological orthogonality). The most appealing example in this case is isotopic labels (with deuterium as a predominant option from a spectroscopic point of view) but also moieties with limited (nitrile, double bonds) or minute biological abundance (e.g., alkynes, azides). Likewise, it is similarly important that the tag is stable within the environment (chemical orthogonality) so that it can be distinguished unambiguously within its spectral environment in the recorded vibrational spectrum (spectral orthogonality). The latter aspect qualifies the silent wavenumber range between 1800 cm^−1^ and 2700 cm^−1^, and thus triple-bond moieties or deuterium labels, where the reduced mass causes the shifting in this unusual wavenumber range, as valid options.

#### Metabolic incorporation and activity probing

Raman tags play an inevitable role in probing the metabolic activity of an organism and identifying regions of highest biochemical turnover as they allow for a distinction between existing and newly synthesized biomass by the appearance of a silent region signal over time. A wide range of substrates have been employed for this purpose to date, many of them also featuring commercial availability. Single-cell applications span major biomolecular classes, including tagged lipids, carbohydrates, amino acids/proteins, and DNA/RNA precursors, to track the most essential metabolic pathways and their efficiency [41]. The use of Raman-tagged derivatives for elucidation of single-cell metabolism has been extensively reviewed in detail elsewhere; we therefore direct the reader to those publications for more detailed elaboration on metabolic pathways [42, 43]. Applying the concept to higher organisms has also been demonstrated successfully, e.g., for fungi [44], live nematodes [45, 46], zebrafish [47, 48], flies [49–51], and even mice [48, 52], where also the use of D_2_O is for some species an efficient strategy to probe the metabolism. Research in this area focuses on metabolic changes caused by, e.g., embryonic development, growth and aging, diseases, and gene effects as well as environmental influences such as microplastic.

In bacterial systems, probing metabolic activity using Raman tags can ultimately reveal the effectiveness of antibiotics on specific strains, usually via D_2_O or deuterated glucose metabolism [39, 53, 54], enabling the profiling of antibiotic resistance (Fig. 2B). As antibiotic resistance continues to rise, there is an urgent need for rapid and reliable screening methods to revolutionize the current approaches in future medical practice, and the Raman-tagged approach can thus help to fill this gap.

Beyond the pathogenic challenges posed by certain bacterial strains, many others play essential roles in environmental systems. Here too, metabolic investigations using silent region Raman tagging can provide valuable insights into how microorganisms respond to and mitigate environmental stressors. A prominent example is their interaction with and potential degradation of (biodegradable) microplastic pollutants [55].

#### Reaction monitoring via the presence of silent wavenumber bands

Although Raman spectroscopy has demonstrated significant potential in reaction kinetics and monitoring [8, 9], the specific application to reactions involving structural motifs within the silent wavenumber region offers considerable untapped opportunities, deserving further exploration. One key example is the Cu-catalyzed azide–alkyne cycloaddition (CuAAC), eventually yielding triazoles under loss of both triple-bond moieties. Click reactions of alkynes and azides are widely used in biorthogonal chemistry, for instance in attaching external fluorescent labels to small molecules [56, 57].

As opposed to investigations using IR spectroscopy focusing on the fate of the azide [58], the alkyne band is much more suited for Raman spectroscopy [40]. Tipping et al. [40] demonstrated this effectively by profiling the kinetics of CuAAC between the Raman probe EdU (5-ethynyl-2′-deoxyuridine) and biotin-PEG-azide (Fig. 2C). The spectral isolation of the alkyne band greatly simplifies the analysis. Variations of this reaction were later also studied by other groups: Tireli et al. [59] used a ball mill approach, encountering partial luminescence interference from copper species, while Scherrer et al. [60] monitored CuAAC on gold surfaces for self-assembled monolayer (SAM) formation via SERS, where plasmonic effects added complexity regarding the visibility of vibrational bands. Other prominent examples involving intrinsic Raman-active alkynes include the Sonogashira cross-coupling reaction and the Glaser-Hay reaction, which will be discussed in the “Reaction monitoring via shifts of silent wavenumber bands” section.

### Raman tags are small(er)

As outlined above, one of the most severe problems of fluorescence microscopy with small molecules is the need for external fluorescent labels. These external labels can become quite large [35] and consequently it is a matter of fact what then actually rules the properties of the small molecular target. Still the target or rather its label? Aside from steric aspects arising from bulkiness which might interfere with trafficking properties of the small molecule, also the retainment of functionality, bioactivity or chemical properties such as hydrophilicity need to be critically reviewed.

**Fig. 3:**
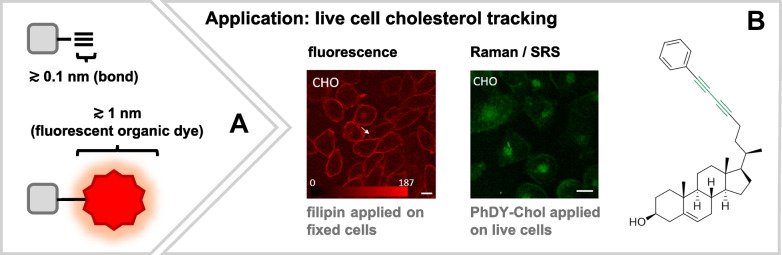
**A** Raman tags are typically only a few bonds in size, making them much smaller than conventional fluorescent labels. As a result, they are less likely to interfere with the native biological behavior of the target, making them well-suited for live-cell imaging. **B** A notable example where Raman tagging can complement or even replace fluorescence imaging is cholesterol visualization: the gold standard dye filipin is limited to fixed cells, whereas Raman tagging enables live-cell analysis. Elements of panel **B** are reprinted from Ref. [62] under CC BY 4.0

Raman tags, in contrast, are small (ideally only a few bonds in size, Fig. 3A) and are therefore considered less disruptive and ideal for live-cell applications. However, the development of efficient tags (see next section) sometimes conflicts with the original goal of minimal structural modification. As such, the resulting properties of tagged molecules must be critically evaluated. This is supported by our own research, where modifications to the sensitive cholesterol scaffold occasionally altered its native distribution [61]. Nonetheless, compromises between highest efficiency and minimal structural modification have proven to be very effective for practical use, thereby serving, e.g., as alternative or complementary imaging strategies to conventional fluorescence imaging. Biosynthetic tagging approaches, such as incorporating tagged amino acids as precursors for peptide synthesis, as demonstrated with the anthelmintic malpinin [28], can further be an effective tool in tag synthesis, acting as an initial proof of biocompatibility. However, such strategies may not be universally applicable.

#### Live-cell imaging

Live-cell imaging is among the most promising real-world applications of Raman tags, given the importance of temporal dynamics in bio(medical) research. Key uses include metabolic pathway analysis (“Metabolic incorporation and activity probing”), drug targeting (“Drug and toxin targeting”), and intracellular tracking via pulse chase experiments (“Classical multiplex imaging”). Combined with fast nonlinear techniques, like SRS and CARS, these tags offer fluorescence-like functionality but with minimal biological perturbation. For examples, see the relevant subsections.

#### Complementary imaging

Speaking of biological gentleness, silent wavenumber tagged substances exploit huge potential in cases where fluorescence imaging is not possible, cannot retain biological functionality, or even requires fixing or where results of fluorescence imaging are suspected to be right. Published examples for these use cases cover, among others, cholesterol Raman probes, with the fluorescent gold standard filipin only operating on fixed cells [61–63] (Fig. 3B), small structure tagging as in nanoparticles where the instability of fluorescence tags can cause uncertainty in the results [64] or where size effects might become critical, affecting, e.g., distribution dynamics. Silent region tagging is thereby not considered a superior solution, but rather complementary, offering even benefits from the interplay of high sensitivity fluorescence and high specificity Raman imaging [28].

### Sensitivity boost concepts

Detecting minute amounts of analytes necessitates substantial enhancement of Raman signal intensities. The intensity of spontaneous Raman scattering $I$ is given by

$$I\propto {I}_{0}N\cdot {{\left(\frac{\partial \alpha }{\partial q}\right)}_{{q=q}_{0}}}^{2}\cdot {\omega }^{4}$$

where ${I}_{0}$ is the intensity of the incident beam, $\omega$ the scattered light frequency, $N$ the number of scatterers, and ${{\left(\frac{\partial \alpha }{\partial q}\right)}_{{q=q}_{0}}}$ the change of the polarizabiliy along the normal coordinate when oscillating around the equilibrium position. This relationship highlights that, from a synthetic perspective, enhancing the Raman signal depends on maximizing the change in polarizability during molecular vibration. While the given expression applies specifically to spontaneous Raman scattering, the change in polarizability is equally critical in its stimulated counterparts CARS and SRS. Therefore, the considerations outlined here are broadly applicable to the design of Raman-active tags across both spontaneous and stimulated modalities.

**Fig. 4:**
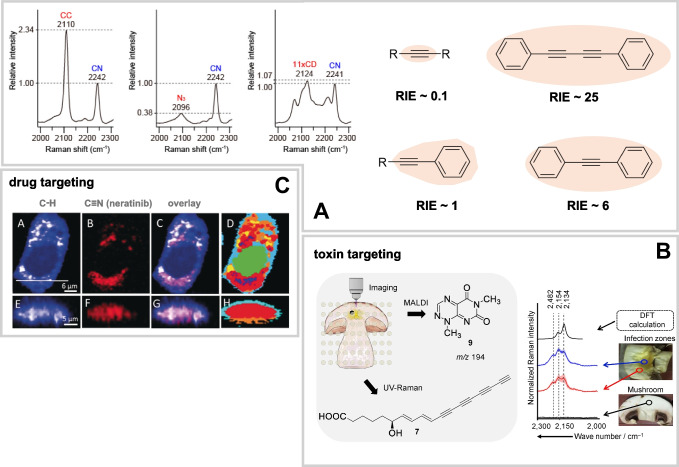
**A** Extensions of the conjugated system enclosing the triple- bond element increase the polarizability and consequently the scattering efficiency of the tag as measured by its RIE. Alkynes usually owe the highest intensities as compared to nitriles, deuterium labels, or azides. Reprinted (adapted) with permission from Ref. [65]. Copyright 2021 American Chemical Society. Inherent tags can be found in nature as part of toxins (here: caryoynencin in infected mushrooms, **B**) or as a part of certain medications (e.g., the tyrosine inhibitor neratinib (**C**)). Permission for reuse of the elements of panel **B** from Ref. [66] has been granted by John Wiley and Sons. Panel **C** was reproduced from Ref. [37] under CC BY-NC-ND 4.0

Polarizability $\alpha$ describes how easily the electron cloud can be distorted by an external electric field (e.g., laser excitation) directly influencing the intensity of the Raman signal. Consequently, the delocalization of electrons, e.g., controlled by the extension of the conjugated π-electron-system around the triple bond in the case of nitriles and alkynes, is a setscrew for the effective design of Raman tags (Fig. 4A). To allow for a fair comparison of different tags, Yamakoshi et al. [65] established a reference-based framework for comparing Raman tag performance, using EdU as a standard. First, it was verified that the relative intensity versus EdU (RIE) indeed increases with the extension of conjugated triple bonds, ranging from an RIE of 0.1 for internal alkynes of the type R–C≡C-R up to 25 for diynes of the type Ar–C≡C–C≡C-Ar [65]. It was further demonstrated that among the available options within the silent wavenumber regions, alkynes perform best, while nitriles provide about three times lower Raman intensity (Fig. 4A) [65]. The efficacy of deuterium tags strongly depends on the number of deuterium atoms involved; however, increasing the number of deuterium atoms typically leads to a significant broadening of the band structure (Fig. 4A).

In addition to employing aromatic and unsaturated systems, the use of lone electron pairs can also significantly enhance polarizability and thus the scattering cross section of a tag, as demonstrated by the Potma group through the inclusion of sulfur adjacent to the alkyne motif [67].

#### Drug and toxin targeting

An application where tag efficiency is critical is drug targeting or toxin sensing, where reliable detectability at native or pharmacologically relevant concentrations must be ensured, as exceeding this critical threshold can severely limit the interpretability of results. While efficient alkyne and nitrile structures are rare in nature (see biological orthogonality), the few natural occurrences of such motifs are fortunate exceptions. Polyyne toxins, which are very suitable for Raman detection, are, e.g., known for bacteria interacting with algae [68] or fungi (Fig. 4B) [66] and are also present in some plants (e.g., falcarinol polyacetylenes in carrots, ginseng and ivy) [69–71] and thus allow for profound investigations into cross-kingdom interactions, action principle, and knockdown mutants, as well as distribution across cultivars.

Few examples exist for tracking labeled drugs in organisms, where drugs are defined as molecules designed for human pharmaceutical use, distinct from biomolecular building blocks discussed in metabolic imaging. Examples include tyrosine kinase inhibitors featuring intrinsic triple bonds, investigated for uptake and metabolism via SRS and spontaneous Raman spectroscopy (Fig. 4C) [37, 72, 73]. Other drugs with intrinsic probes, like ethinylestradiol (contraception) and rilpivirine (HIV), remain largely unexplored by spontaneous or stimulated Raman scattering. External tagging is possible but rarely reported, likely due to the low drug concentrations that challenge detection without signal enhancement.

### Band shape and position

**Fig. 5:**
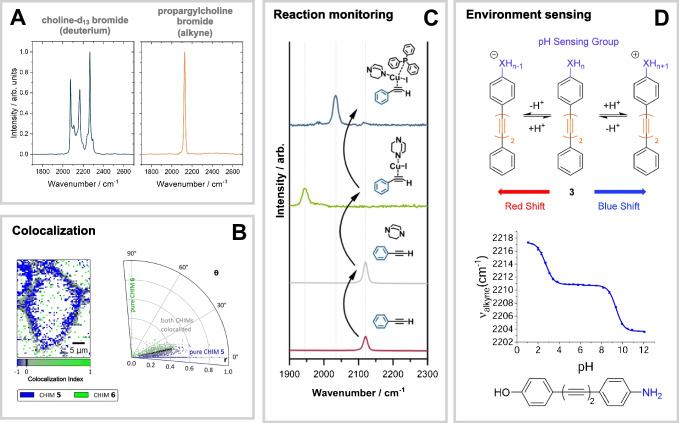
**A** Comparison of the band shapes of deuterated and alkyne-tagged choline bromide (own data). **B** Different tag designs result in distinct Raman shifts. Comparing the relative intensities of two tags in multiplexed applications enables colocalization analysis and assessment of the quality or similarity of the mimetics. Reproduced (adapted) from Ref. [61] under CC BY 3.0. **C** The electronic properties of a tag can be tuned to shift its spectral position, which is useful for elucidating reaction mechanisms (e.g., Sonogashira coupling). Reproduced (adapted) from Ref. [75] under CC BY-NC 4.0. **D** Environment changes around the tag (such as pH) can influence its spectral features, allowing alkynes to serve as chemical sensors. Elements reproduced from Ref. [76] under CC BY 3.0

A standout advantage of fluorescence tags is their ability to distinguish multiple species simultaneously via distinct emission maxima. Similarly, in Raman-based applications, the placement of bands within the silent wavenumber region is an important parameter for multiplexing applications. Raman bands are inherently much narrower (FWHM ≈ 10 cm^−1^) than typical fluorescence emission bands (FWHM of several nanometers), making them well-suited for multiplexing purposes when well-separated. However, beyond width, the shape of a Raman band ultimately determines its practical utility. The peak shape largely depends on the number of active Raman modes with bands in the spectral region of interest. For single nitriles and alkynes, the Raman-active mode typically involves a single stretching vibration (Fig. 5A), resulting in a sharp, well-defined band, making them consequently a suited choice. In contrast, deuterium labels present a more complex case. When incorporated into biomolecules, deuterium can influence multiple Raman-active modes, such as symmetric and antisymmetric C–D vibrations (Fig. 5A), or in certain cases by H/D exchange, leading to broader or more heterogeneous bands [74], which limits their effectiveness in multiplexing applications.

Tuning the position of an alkyne or nitrile marker band within the spectroscopically silent region requires structural modifications. A widely used strategy involves extending simple alkynes to diynes or polyynes, enabling the creation of tag libraries with finely adjustable vibrational frequencies [33, 77]. These frequency shifts can be achieved by modifying the conjugation length [33], substitution pattern, and choice of substituents [33, 65, 77], or through incorporation of isotopic labels [33, 78, 79]. While diynes and polyynes can generate more than one Raman band within the silent wavenumber region [33, 61, 68], experimental observations often reveal only one dominant band, which justifies their use as tags with multiplexing capability [33, 61, 65, 77]. For the cholesterol-diynes studied by Schultz et al. [61], the spectrum was, e.g., dominated by a symmetric vibrational mode in which both alkyne units moved in concert, as resolved by quantum chemical calculations. Consequently, the band patterns of di- and polyynes arise from collective motions of the yne-framework, as defined by the molecule’s normal modes [61, 68].

Normal modes describe the characteristic patterns of atomic motion within a molecule, in which atoms oscillate with the same frequency and a fixed phase relationship. The vibrational frequencies associated with these normal modes depend on both the bond strengths and the effective vibrating mass of the involved atoms. For a simple diatomic system or for isolated single bond vibrations, the vibrational frequencies can be compared based on

$$\nu \propto \sqrt{\frac{k}{\mu }}$$

where *k* describes the bond strength and $\mu$ the reduced mass of the vibrating masses.

In more complex systems, such as conjugated chains or substituted polyynes, predicting vibrational frequencies becomes more difficult due to coupled atomic motions. In such cases, density functional theory (DFT) calculations are typically required for accurately resolving the nature of the vibrational modes and a reliable prediction of the origin of design-caused effects on the vibrational frequency.

Regarding the applications of Raman tags discussed in this chapter, Raman-based strategies generally fall into two complementary categories: one aims to engineer molecular structures that shift vibrational bands for multiplexed detection (e.g., barcoding and colocalizing), while the other interprets spectral shifts to infer underlying structural changes (reaction monitoring and sensing).

#### Classical multiplex imaging

Arising from the probing of metabolic activity, incubation with tagged substances allows highlighting the locations of highest metabolic activity. Consequently, if the probes are chosen strategically, certain cell organelles can be highlighted as followed from their function. Probes initially developed for assessing metabolic activity can frequently be repurposed for specific organelle labeling, enhancing their utility for multiplexed imaging; e.g., tagged nucleotides for visualizing the nuclei, tagged lipids for the investigation of lipid droplets, among others that are specially designed for mitochondria or lysosomes [80, 81]. Targeting specific organelles is particularly important when evaluating the influence of an external disturbance (e.g., a disease state) [82]. In other cases, they could be used as references for probing the functionality of an unknown tagged analyte. Colocalization analysis with established probes can provide insights into the analyte’s behavior by revealing similarities in spatial distribution and potential functional association [61].

In the context of optimized multiplexed detection, tags and targeting groups are often treated as modular units. This separation enables the development of versatile palettes of efficient Raman tags that can be custom-tailored and linked to specific targeting moieties to direct their localization [33, 80]. However, it remains essential to prioritize minimal tag size to preserve biological function and minimize steric interference. While the simultaneous visualization of commonly studied organelles as well as their number would often be feasible via fluorescence imaging, the flexibility in tag placement offered by multiplexed Raman probes with narrow bandwidth provides a distinct advantage and less limitation. Furthermore, organ tracking by multiplexed Raman not only enables tailored targeting but also preserves the ability to extract chemical, label-free information from the surrounding environment, an insight that is far more challenging to obtain using fluorescence alone or even combined Raman-fluorescence methods. Building on this foundation, newer generations of Raman tags are being engineered for enhanced functional versatility. For instance, a photoactivatable class of tags was introduced by Du and Wei [83]. While such tags may not be functionally necessary in many applications, they may offer strategic advantages in scenarios where spectral overlap between tagged substances occurs, eventually enabling temporally staggered probing of analyte distributions [83]. Reversibly switching Raman probes [84] further allow for pulse chase experiments to visualize dynamic intracellular processes like fission, fusion, and dynamic motion.

#### Barcoding

While in classical multiplexing applications each Raman probe targets a single analyte, barcoding leverages combinations of a limited set of spectrally distinct probes mixed in defined ratios. This approach generates unique spectral intensity patterns that serve as barcodes, often allowing the number of identifiable codes to exceed the number of individual tags. For a number *r* of spectrally distinguishable Raman tags with narrow single bands and *i* assumed intensity levels (starting from 0) for each code digit, the number of overall codes *N* evaluates to $N={r}^{i}-1$. Assuming that no reliable absolute values can be distinguished but relative values matter, the number of possible codes is reduced, while it needs to be reminded that different RIE values of the tags can further eliminated codes from practical use. The use of Raman probes within polymer beads for barcoding applications was suggested by the Wei Min group in 2018 in context of the Carbow-palette [33]. Later, barcodes were created internally by incubating different cell lines with Raman-dots bound to surface proteins, ultimately leading to cell profiling as a cause of different expression levels [85]. Aside from these two examples, no applications where such alkyne-barcoded probes were used in a real-world applications example could be found. Although the narrow bandwidth of Raman probes makes them ideal for barcoding applications, we believe that their competitiveness with conventional biological methods is still too weak. Position-based barcoding still rules assay analysis, requiring new methods to match its speed and infrastructure compatibility [34], while alternative tag-free approaches are competitive in applications like cell profiling [86, 87].

#### Reaction monitoring via shifts of silent wavenumber bands

Following the example on reaction monitoring, there are also reactions in which the alkyne unit is not consumed but also appears in the product. Sonogashira coupling, transforming primary alkynes into secondary alkynes, and its possible side reaction Glaser-Hay homocoupling, transforming alkynes into diynes, are examples thereof. These reactions are interesting for Raman monitoring as in both cases the substitution pattern of the alkyne is changed, leading necessarily (Glaser-Hay-coupling) or possibly (Sonogashira with aryl halides) to an increase in length of the conjugated system. Consequently, bands at higher wavenumbers appear, indicating the successful synthesis of the product [40, 75]. In the case of the Sonogashira coupling, Pickardt et al. [75] observed that different states of coordination could be additionally distinguished (Fig. 5C). Specifically, side-on complexation of the alkyne to Cu(I) reduces the alkyne’s electron density, thus weakening the bond. Consequently, the C≡C stretching vibration shifts to lower wavenumbers (2125 cm^−1^ → 1940 cm^−1^/2035 cm^−1^). The exact shift depends on the electron density of the triple bond, with the Cu center acting solely as an electron-withdrawing partner and not partaking directly in the vibration, and thus ultimately on the degree of substitution on the metal center [75].

#### Sensing

Closely related to reaction monitoring is the topic of sensing, i.e. employing silent region-tagged molecules as an optical indicator for either the surrounding environment or the presence of another molecular class whose identification would be otherwise much more difficult.

pH-sensitive probes were reported by the Sodeoka lab in 2014 on nitrile [88] and the Graham and Tomkinson labs in 2020 and 2021 for alkyne-based [76, 89] structures. These reported tags possess an acidic hydrogen atom and, depending on the pH of the environment, the equilibrium between deprotonated and protonated forms can be shifted. Deprotonation thereby creates a lone electron pair extending the system of delocalized electrons and ultimately changes the sensors’ structure (Fig. 5D). Consequently, the bands in the silent wavenumber region of protonated and deprotonated forms are distinct from each other. For intercellular and live-cell applications, sensors with a *pK*_*a*_ around 6 come on hand and were successfully employed, with expectable shifts in the spectrum arising within the relevant pH window in cells of 5–7 [76, 88, 89]. However, it has also been shown that by varying the substituents on the BADY-Ph rings, BADY-based tags can be tailored to operate across a *pK*_*a(H*)_ range of 2.7 to 9.7 for diverse applications [76]. While pH is likely the primary driver of structural changes, environmental factors such as solvent polarity and proton-donating ability can also stabilize one form over the other. For example, the protonated form of the nitrile sensor FCCP predominates in pure aprotic DMSO [88].

Raman tags have been also used to monitor H/D exchange, with terminal alkynes acting literally as "tagged tags" with an alkyne band shifting > 100 cm^−1^ upon deuteration; the simultaneous loss and gain of C–H and C–D bands revealed exchange kinetics [90].

Raman tags can also serve as sensors for other molecules, with one example presented by Yamakoshi et al. [91]. Here, the presence of thiols is detected by a reaction with a Raman-tagged Michael acceptor (cyanoacrylic acid) eventually yielding an alkyl nitrile with a glutathione substituent. The structural change of the sensor causes a spectral shift of 20 cm^−1^ towards smaller wavenumbers and was even suitable for use in cells [91].

Having discussed the key aspects and diverse applications in detail, we summarize essential considerations for effective Raman tag design in Box 1.

**Box 1** Key considerations for Raman tag design.

**Table:** 

Orthogonality	Chemical and spectral distinctness from the environment
Minimal size	Ensuring biological compatibility and minimal functional interference
Signal enhancement	Maximizing polarizability for optimal Raman intensities
Multiplexing capability	Narrow and distinct spectral bands enabling simultaneous detection of multiple analytes
Environmental sensitivity	Capability for sensing environmental parameters (e.g., pH, H/D exchange, reaction states)

## Conclusion and outlook — towards single molecule detection

This review has highlighted the evolving role of Raman tags in both spontaneous and stimulated Raman scattering, with a focus on bioanalytical applications. During review of the literature, we recognized that Raman tagging approaches are nowadays often closely related to stimulated modalities, particularly SRS. While time-sensitive scenarios like live-cell imaging or large-area screening clearly demand such fast imaging methods, in cases where temporal resolution is less critical or detailed hyperspectral information is required, spontaneous Raman remains a powerful tool (and not an alternative) due to its rich spectral content. Recent advances in broadband CARS and SRS promise to bridge the gap between speed and spectral richness, potentially enabling real-time, chemically detailed imaging. Such capabilities could transform applications into real-time diagnostics, live-cell metabolic studies, and rapid environmental sensing using Raman tags.

Despite advances in fast imaging, the low concentrations typical of many biological systems continue to challenge the sensitivity of Raman-based methods. In such cases, there is often no practical benefit from using fast techniques like SRS, as the inherently slow sampling required for sufficient signal accumulation negates their speed advantage, and spontaneous Raman could thus be more effective. To overcome these limitations, the development of efficient and minimally invasive Raman tags, especially for small bioactive molecules, remains a priority. Current strategies, such as linker-based designs inspired by fluorescence tagging, often conflict with the principles of Raman tag design, while precursor-based approaches frequently suffer from low scattering efficiency.

Emerging methods like stimulated Raman excited fluorescence (SREF) and pre-resonance excitation [92–94] offer significant sensitivity enhancements. However, current limitations concerning tag size, complexity, and biocompatibility must be overcome to realize their full potential in practical applications. Nonetheless, SREF represents a compelling alternative to fluorescence, offering enhanced chemical specificity without sacrificing detection sensitivity. Looking forward, concepts such as photoswitchable or switchable Raman tags, potentially enabling super-resolution analogs to STED in Raman imaging, could further extend the frontiers of the field.

Beyond traditional bioanalytical contexts, we foresee a broader adoption of Raman tagging strategies in environmental monitoring and materials science. Applications such as reaction tracking in smart materials (including shape-memory and self-healing polymers) could represent promising new directions. But aligned with global sustainability goals (e.g., Agenda 2030), the continued refinement of Raman tagging technologies will likely prove critical across diverse scientific domains.
